# Neural tracking at theta predicts drumming-induced altered states of consciousness

**DOI:** 10.1038/s41598-026-37700-x

**Published:** 2026-03-26

**Authors:** Yoel Gordon, Golan Karvat, Noa Dagan, Ayelet N. Landau

**Affiliations:** 1 Department of Cognitive Sciences, Hebrew University of Jerusalem, Jerusalem, Israel; 2 MRC Cognition and Brain Sciences Unit, University of Cambridge, Cambridge, UK; 3 Department of Experimental Psychology, and Division of Psychology and Language Sciences, University College London, London, UK; 4 Max Planck School of Cognition, Leipzig, Germany

**Keywords:** Consciousness, EEG, Auditory perception, Altered states of consciousness, Neural entrainment, Neural rhythmic tracking, Rhythm perception, Time perception., Neuroscience, Psychology, Psychology

## Abstract

Altered states of consciousness (ASC) involve shifts in perception, cognition, and awareness, offering a unique lens on the nature of consciousness. Across cultures, rhythmic drumming in the theta range (4–7 Hz) is often used to induce such states, but it remains unclear whether actively enhancing brain rhythms in this range can cause ASC. We tested whether theta-frequency drumming (4 beats per second) selectively induces ASC compared to rhythms in the delta and alpha ranges. Forty participants listened to ten-minute drumming sessions at each rhythm while EEG was recorded. After each session, they completed an altered experience questionnaire and a time estimation task - an implicit ASC measure. Rhythmic neural tracking at theta was linked to stronger altered experience. Furthermore, participants with stronger theta-band neural tracking to 4-bps drumming tended to report higher ASC across all drumming rhythms, suggesting that neural tracking at this rhythm might serve as a neural marker of susceptibility. These rhythm-specific effects indicate a bidirectional relationship between theta activity and ASC: not only do theta activity accompany ASC, it may actively modulate it. These findings shed new light on the neural dynamics of consciousness and suggest that rhythmic stimulation could serve as a non-pharmacological method for modulating conscious states, with potential applications in cognitive neuroscience and therapeutic contexts.

**Supplementary Information:**

The online version contains supplementary material available at 10.1038/s41598-026-37700-x.

## Introduction

Our subjective experience comprises various states of consciousness beyond everyday waking awareness such as dreaming, meditation, and states induced by several psychoactive substances. These states, collectively referred to as Altered States of Consciousness (ASC), can be evoked through techniques such as dancing, consuming psychoactive substances (e.g., psilocybin), chanting, sensory deprivation, and listening to repetitive rhythmic drumming^1^. Among these, repetitive rhythmic drumming is considered one of the earliest and most wide-spread and effective methods of ASC induction^2,3^. Across cultures, drumming often serves as the sole element in rituals aiming to evoke ASC^4^. In such rituals, a defining feature of drumming used to induce ASCs is a repetitive, monotonic rhythm typically ranging from 4 to 9 beats per second (bps)^5–7^. Such rhythms often elicit trance states, characterized by narrowed external awareness, altered thinking, time distortions, loss of physical control, perceptual changes, and vivid imagery^8^. The cross-cultural similarities in the rhythms used to evoke these mental states indicate a possible engagement of a common biological basis.

The rhythmic nature of drumming-induced altered states of consciousness (ASC) suggests that their neural underpinnings are tied to the brain’s intrinsic rhythmic activity - neural oscillations. These rhythmic patterns of neural activity are not only fundamental to cognitive and perceptual processes^9^, but are also associated with distinct conscious states. This association is exemplified most clearly in the progression through sleep stages^10^. The ritualistic drumming at 4–9 bps align with these intrinsic oscillations, particularly within the theta band (4–8 Hz), which has been associated with ASC such as deep meditative state^11,12^, and hypnosis^13^. In these contexts, neural entrainment may occur, wherein the brain’s intrinsic oscillatory activity synchronizes with external rhythmic stimuli, such as drumming. Neural entrainment has been widely studied for its effects on behavior^14–19^. However, little is known about the influence of neural entrainment on subjective experience^20,21^. Previously, theta-range entrainment was proposed as a mechanism that promotes the characteristic perceptual and experiential shifts of ASC^6^. While ASC has been linked to increases in endogenous theta activity, it remains unclear whether externally driven theta (e.g., in response to drumming) actively evokes ASC. Demonstrating that externally driven theta, in addition to internally generated theta, is associated with increased ASC would provide critical insights into the neurophysiological mechanisms underlying these states. The present study will investigate whether externally driven theta is associated with ASC. Although such an association could serve as evidence for the original entrainment proposal, i.e., that neural entrainment of endogenous theta is driving the ASC, we maintain a broader interpretive stance. Specifically, we remain agnostic as to whether the observed theta-rhythmic neural response reflects modulation of intrinsic oscillatory activity or a sequence of externally evoked responses. Thus, we will refer to this brain response as neural tracking from here on when referring to our measures and results.

Previous studies that investigated the role of externally driven theta in ASC reported inconclusive results. For instance, Maxfield^22^ found that monotonic drumming at 4.5-bps increased theta activity and evoked trance-like experiences, including sensations of flying and temporal distortions. In contrast, Konopacki^23^ reported no significant increases in theta activity or ASC-related experiences in response to 4-bps drumming, even after incorporating trance-inducing instructions. Similarly, Hove et al.^24^ found no difference in theta activity between trance and no-trance conditions in experienced western shamanic practitioners. Lastly, Huels et al.^25^ used a similar drumming stimulus and observed increases in gamma power (30–45 Hz) with trance-related 4-bps drumming, but no significant changes in theta was found. These inconsistencies may reflect limitations in prior analyses. For example, these studies relied on absolute power measures, which quantify the total power within a specific frequency band but do not differentiate between rhythmic oscillatory activity and non-oscillatory features. Although often used in spectral analysis of neural studies, power-based measures only provide a partial insight to processes of neural tracking. To best characterize it, phase-based measure or synchronization between brain rhythms and external rhythmic stimuli is required^26^. Furthermore, the susceptibility of power-based measures to the non-oscillatory spectral features (i.e., 1/f, the diminishing power values with increased frequency) can obscure oscillatory activity at specific frequencies, and make comparisons between different frequency bands difficult^27,28^. Specifically, the absence of an evident theta-rhythmicity increase in response to a strong external entrainer in this rhythm, as seen in Konopacki^23^ and Huels^25^, further highlights the need for more robust methodological frameworks for capturing neural rhythmic tracking.

To address these limitations, our study employs advanced phase-based measures over extended time windows, a larger sample size, and data-driven electrode choice (rather than relying on a prior assumption) to identify relevant electrodes responding to the rhythmic drumming, and better capture the neural dynamics underlying ASC. In addition, another explanation for these inconsistencies is the great individual variability in susceptibility to ASC^29^. Although some individuals readily enter altered states, others experience little to no change. Previous research has primarily sought to explain this variability through factors such as personality traits (e.g., absorption or openness to experience), neuro-structural characteristics (e.g., corpus callosum morphology), psychopathological conditions, or behavioral tendencies^29–33^. However, the role of variability in neurophysiological responsiveness to the external stimuli in ASC susceptibility has yet to be explored.

In this study, we use EEG to examine whether neural rhythmic tracking to drumming at theta is associated with stronger ASC-related experiences. We examined theta rhythms, since it is commonly used in traditional contexts and compared both experience and neural responses to this rhythm to higher and lower drumming rhythms. Forty participants were immersed in three 10-minute isochronous drumming epochs while EEG was measured. Each drumming epoch had one of three rhythms (1.5-bps, 4-bps, and 9.5-bps) corresponding to delta (1–3 Hz), theta (3–8 Hz), and alpha (8–12 Hz) bands, respectively. This design enables the examination of whether altered states of consciousness are uniquely associated with neural tracking at theta-range, rather than with rhythmic tracking in general. After each drumming epoch, participants completed an altered experience questionnaire (a questionnaire inspired by an abbreviated version of the Phenomenology of Consciousness questionnaire - PCI^34^, see "Methods") to assess subjective experiences. Furthermore, the participants estimated the drumming duration to assess distortions in time perception, a prominent feature of ASC, and has been shown to change to unconscious changes in cortical arousal^35^.

We hypothesized that 4-bps drumming would elicit the strongest ASC-related experiences, reflected in higher questionnaire scores and more distorted time estimations. On a neural level, we predicted a rhythm-specific effect, where stronger neural rhythmic tracking at theta, quantified by EEG-to-stimulus coupling, would correlate with intensified subjective experiences of ASC. We also hypothesized that this coupling would correlate with time estimation. If such a rhythm-specific relationship were confirmed, it would provide compelling support for the idea that the traditional use of theta-range rhythms in ritual practices is grounded in distinct neurophysiological mechanisms. This study thus aims to bridge cultural practice with modern neuroscience by rigorously testing whether the 4-bps rhythm, often used in ceremonial contexts, holds a unique role in facilitating ASC through measurable neural tracking.

## Methods

### Participants

Forty participants (26 females, 14 males, mean age = 24 years, SD = 3.07) took part in the study. All participants were students at the Hebrew University of Jerusalem and were recruited via the university’s online experiment recruitment system. Participants were unexperienced in ASC and were naïve to the experiment’s goal. Inclusion criteria included no diagnosis of ADHD or hearing impairments. In the analysis, one participant was excluded from any within-subject analysis of time estimation analysis due to a technical error in data collection in one of the blocks. Furthermore, one participant was completely removed from the analysis since he reported that he often fell asleep during the experiment.

All participants provided written informed consent before the experiment. The experiment was carried out in compliance with the Declaration of Helsinki and was approved by the IRB ethics board of the Hebrew University of Jerusalem.

### Design and procedure

After providing informed consent, participants were fitted with a 64-electrode EEG cap (see EEG recording section). They were then seated comfortably in an armchair in front of a computer screen. The experiment began with a brief introduction, designed to help participants relax and focus on their inner experience, after which they were left alone for 3 min. Then, the main experimental procedure, operated by a custom-built Python based program began. During the trials, participants were requested to close their eyes and wear an eye cover, to further block any external visual stimuli.

**Fig. 1:**
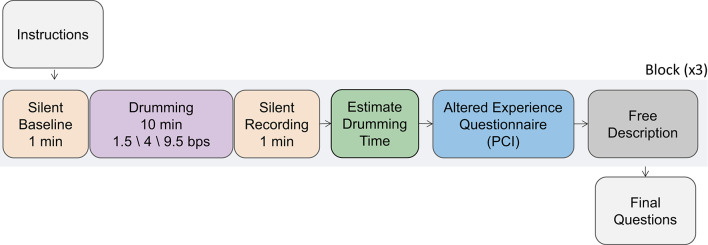
Experimental procedure. Participants listened to three different rhythmic drumming blocks at different rhythms (1.5, 4 and 9.5 bps) while EEG was recorded. Each 10-minute-long drumming epoch had a 1-minute-long silent baseline EEG recording before (S1) and after (S2) the drumming. After each block, participants estimated the drumming duration, filled an altered experience questionnaire and freely described their experience. Further personal details were collected at the end of the experiment (after the third drumming block). The experimental procedure (shaded in gray) was repeated three times, once for each drumming condition.

The experiment had a within-subject design, in which each participant was exposed to three 10-minute drumming stimuli at 1.5, 4, and 9.5 beats per second (bps). These were crafted on the computer to ensure rhythm precision while keeping an ecological sound (see auditory stimuli section). The rhythms were chosen to correspond to the delta, theta, and alpha frequency bands, while avoiding harmonic relations between them (e.g., 8 Hz would be a harmonic of 4 Hz). The rhythmic conditions were counterbalanced using a Latin square design to ensure that each rhythm appeared equally in all positions, either first, middle, or last. Before and after each 10-minute drumming block, a 1-minute silent recording (S1 and S2, respectively, see Fig. 1) was introduced. The S1 section served as a baseline recording of the neural activity before each drumming stimulus. During the whole block (comprising of S1, drumming, and S2 sections), participants were instructed to sit quietly and minimize any movements. Each section in the block was marked by a 2-second-long auditory cue (beep) at the beginning and end to help participants orient themselves. The end of the S2 section was marked by a longer 4-second-beep, signaling the end of the block, at which point participants were free to move, open their eyes, remove the eye cover, and call the experimenter.

After each block, participants estimated the duration of the drumming stimulus using a designated text box on the screen. The experimenter then entered the room, opened a digital version of the altered experience questionnaire (see altered experience (AE) questionnaire section), and left the room again while participants completed the questionnaire privately. Participants were then invited to freely describe their experiences in a text box provided at the end of the questionnaire. At the end of the experiment, the participants answered further questions regarding their general experience in the experiment, and their prior music experience and spiritual practices.

### Altered Experience (AE) Questionnaire

To assess ASC related altered subjective experience we used a tailored self-report questionnaire. This questionnaire was inspired by the Phenomenology of Consciousness Inventory (PCI)^34^. For the present study, we composed the questions to focus solely on altered subjective experience, inspired by items measuring this dimension in the PCI questionnaire (which encompasses additional dimensions). This was done to minimize experiment length, given the extended setup time required for the EEG recording, and to simplify the process of completing the questionnaire multiple times. The tailored questionnaire was composed originally in English, and was translated into Hebrew using a ISPOR^36^ translation procedure: (1) three independent translations into Hebrew were made, (2) discrepancies were discussed and revised, (3) a fourth individual translated the final Hebrew draft back into English, (4) any remaining differences between the original and back-translated drafts were resolved and (5) the questionnaire was tested using a non-EEG pilot. Internal consistency for the questionnaire was evaluated using Cronbach’s alpha, yielding a coefficient of 0.88 (CI = 0.84–0.90), indicating good reliability in our sample.

### Time estimations

Immediately after each drumming block, participants estimated the duration of the drumming section as precisely as possible, including decimal values. All drumming sequences were exactly 10 min long, though participants were not informed of this. To avoid biasing their estimates, no clocks were visible on the screen during the experiment, and participants were asked to remove any time-keeping devices beforehand. Given that time distortion is a well-documented feature of ASC, we hypothesized that time estimations could serve as an implicit measure of ASC, complementing the explicit Altered Experience (AE) scores from the questionnaire. To our knowledge, this method has not been previously applied in ASC research.

### Auditory stimuli

The drumming stimuli were produced using Chromaphone 3 by AAS for in Ableton 11, a plugin specialized in creating realistic percussion sounds. It allows for the replication of various drum membranes, and incorporates dynamic randomization of hit intensity and position on the membrane to simulate real drumming. We used an animal-skin-like membrane preset with randomized intensity and hit position, mimicking the sound dynamics of large ritual drums, while keeping the rhythm monotonous. This digital approach ensured that the rhythms were highly precise, which was crucial for our experiment since rhythm was the primary variable, while keeping an ecological sound. Furthermore, it allowed for a similar pitch despite the changes in tempo across the drumming conditions (compared to simply speeding up or slowing down a drumming recording).

### EEG recording

EEG was recorded using a standard 64-channel passive electrode BrainCap and g.HIamp system amplifier by g.tech with an additional 4 EoG channels to facial muscle activity (2 horizontal and 2 vertical). Two reference electrodes were located on the earlobes. Electrode impedance was ensured to be below 5-kΏ prior to recording. The electrodes were connected to a g.HIamp amplifier, and recordings were performed at a sampling rate of 512-Hz using a Simulink model in MathWorks MATLAB. The Simulink model included a trigger channel, which received auditory information through a trigger box (g.trigbox), and included information on every beat of the drum, as well as triggers signaling the beginning and end of each section in the trial.

### EEG preprocessing

Data analysis was performed using the Fieldtrip toolbox^37^ in MATLAB. First, all electrodes were re-referenced to the average of the two reference electrodes placed on the earlobes. We used the fieldtrip toolbox function ft_preprocessing in order to remove line noise at 50 Hz and its harmonics (e.g., 100 Hz) with a notch filter, high-pass filter at 0.5 Hz. Muscle artifacts were removed using an automated high-amplitude Z-value artifact detection (Fieldtrip’s ft_artifact_zvalue function) followed by a manual inspection and further artifact removal. During manual inspection, bad channels were identified and later interpolated using data from neighboring electrodes (ft_channelrepair). Later, an Independent Component Analysis (ICA) was applied to further clean the data from any eye-movements or blinking components. These were minimal due to the experimental setup, which included blindfolding participants, instructing them to remain still, and having them keep their eyes closed during recordings.

**Fig. 2:**
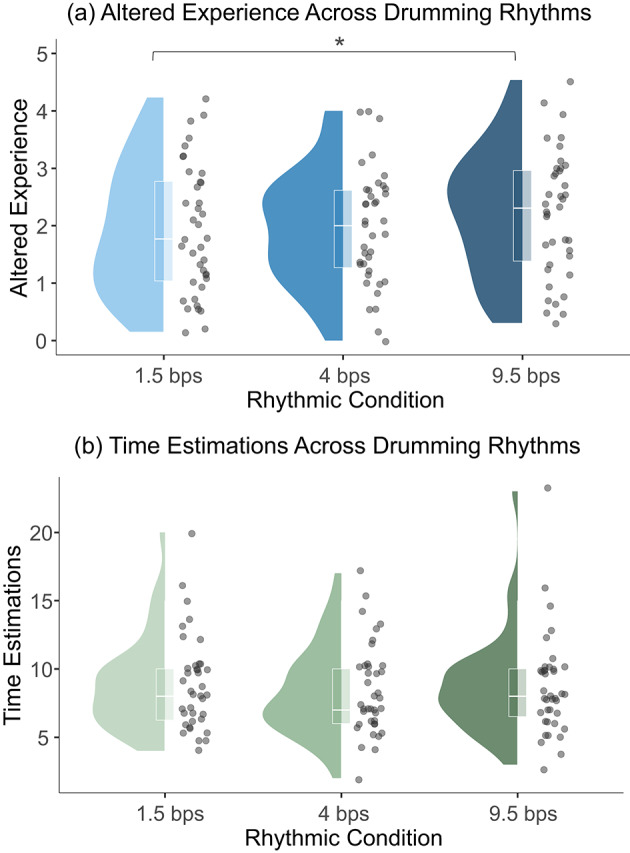
Subjective experience results. **a** Altered experience (AE) scores across drumming conditions. These were assessed using a tailored self-report questionnaire. Participants reported significantly higher AE during 9.5 bps drumming compared to 1.5 bps (t(38) = 2.43, *p* = 0.02). **b** Time estimation results across drumming conditions. Time estimations of the drumming duration were collected after each drumming session to asses distortion in perception of time. No significant differences were observed in participants’ time estimations across conditions.

Electrodes of interest for the analysis were selected using a data-driven cluster analysis approach. EEG rhythmicity (LAVI, see next section) during the drumming epoch was compared to the EEG rhythmicity during the preceding silent baseline (S1) for each drumming condition (1.5, 4, and 9.5-bps). Activity differences were examined at the frequencies corresponding to the drumming rhythms (e.g., 1.5 Hz during 1.5-bps) using cluster-based paired t-tests with Monte Carlo permutation testing with 1,000 iterations^38^. Significant positive clusters, which mean significantly higher rhythmic activity during the drumming compared to S1, were identified at 1.5 and 4 Hz in central-frontal electrodes (*p* < 0.01, see Fig. 2). AT 9.5 Hz, only significant negative clusters were found, indicating a decrease in rhythmicity at 9.5 Hz during the drumming compared to S1 across almost all electrodes. From the significant positive clusters at 1.5 and 4 Hz, we selected electrodes that overlapped between the conditions and had the highest t-values. This resulted in the selection of 10 frontocentral electrodes for further analysis: F3, F1, Fz, F2, F4, FCz, FC2, FC4, AF3, and AF4. These electrodes are consistent with previous literature, where frontocentral electrodes exhibit maximal auditory-driven EEG activity^39,40^.

### Quantifying EEG rhythmicity

**Fig. 3:**
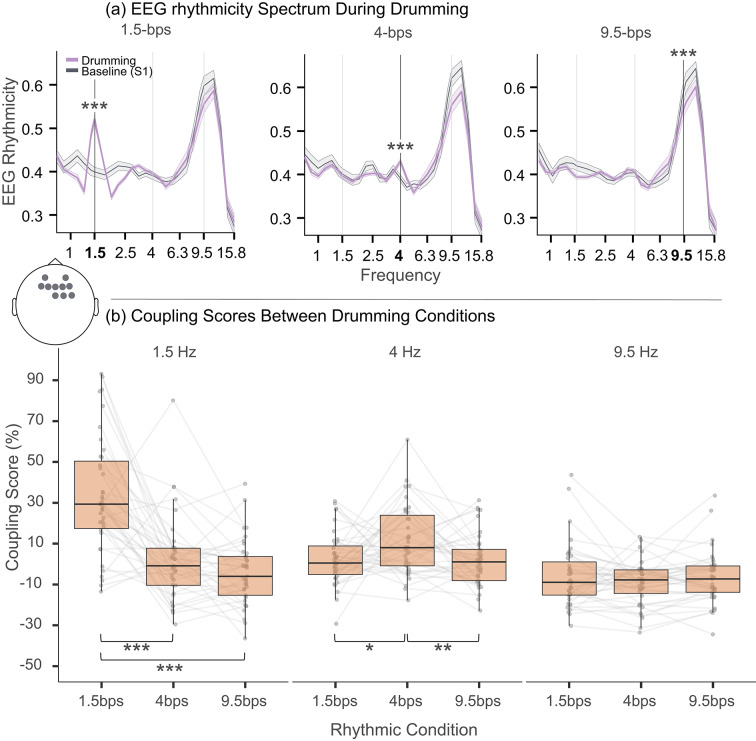
Measuring rhythmicity spectra and neural tracking to 1.5, 4 and 9.5 bps drumming conditions. **a** EEG rhythmicity spectrum (LAVI; line) and standard error of the mean (shaded area) during each drumming condition (purple) and the preceding silent baseline period (S1; gray). Left, middle, and right panels depict data from 1.5, 4, and 9.5 drumming conditions respectively. t-test significance comparing drumming vs. baseline S1 LAVI scores is shown for each relevant frequency. **b** Coupling score within each frequency band (1.5 Hz, 4 Hz, and 9.5 Hz). For each frequency coupling is compared across the three drumming conditions. Data included from channels highlighted in the scalp topography, based on cluster analysis (see Methods). Stars signify significance as follows * *p* < 0.05; ** *p* < 0.01; *** *p* < 0.001.

EEG rhythmicity was quantified using the Lagged Angle Vector Index (LAVI) algorithm^26^. This method quantifies how sustained an oscillation is over a period of time. The foundational principle is that the phase of a genuine, sustained oscillation at any given time point should be predictable from its past and future phase. Thus, it measures the consistency of phase differences between the time series data at a given frequency, and a lagged version of itself, by computing the vector mean of the phase-differences of each data time-point and its lagged counterpart. The LAVI value is the length of the resulted mean vector, with values ranging from 0 for signals with completely random phases to 1 for completely rhythmic signals. This analysis is repeated for each frequency of interest to yield the rhythmicity profile (see Fig. 3a). Thus, a higher LAVI value indicates a more sustained rhythmic oscillatory activity in a specific frequency. The LAVI method is uniquely suited to capture rhythmic tracking for several reasons. First, the mean of the rhythmicity profile across frequencies is stable across datasets^26^ and depends only on the experimentally-chosen lag duration and time-to-frequency-transform method. Importantly, it is robust to the influence of spectral biases, such as aperiodic 1/f components, thereby allowing clearer comparisons across frequency bands at the population level. And second, it is a computationally efficient implementation of the lagged-coherence method^41^ and is therefore well-suited for analyzing rhythmicity over long-duration epochs.

We quantified rhythmicity using LAVI separately for drumming epochs as well as for a silent baseline, S1, preceding the drumming line (S1). To transform the time-domain signal to the frequency domain, a five-cycle wavelet was used. LAVI was then computed with a 1.5-cycle long lag, for 27 logarithmically spaced frequencies between 0.8 Hz and 15.8 Hz. We ensured the inclusion of the three target frequencies corresponding to the drumming rhythms (1.5, 4 and 9.5 Hz) in the spectrum. For each section and condition, we extracted LAVI values at the three frequencies of interest. This resulted in a rhythmicity index for each participant, condition (1.5-bps, 4-bps, 9.5-bps), section (S1 and drumming), and frequency (1.5, 4, 9.5 Hz). In each condition, one frequency matched the drumming rhythm (e.g., 1.5 Hz in the 1.5-bps condition), while the other two frequencies were incongruent (e.g., 1.5 Hz in the 4-bps and 9.5-bps conditions). Notably, a different pattern emerged at 9.5 Hz compared to the lower frequencies. Robust endogenous alpha activity (8–12 Hz), commonly present during a relaxed eyes closed situation, produced strong LAVI peaks in this range. This alpha rhythmicity was consistent across participants and appeared independent of the drumming rhythm, potentially masking any tracking effects from the 9.5-bps stimulation.

In addition to the main analyses, applying the newly developed rhythmicity measures, we also include more commonly used spectral decomposition and inter-trial-phase coherence to demonstrate neural tracking in delta and theta (see Supplementary Figs. 1, 2).

### Coupling scores

To assess neural rhythmic tracking, we computed a measure we term “coupling score”. This score assessed the change in EEG rhythmicity spectrum during drumming compared to the baseline (S1). Although an additional silent period was included following the 10-minute drumming block (S2), we could not include this epoch as a baseline seeing that this epoch might be influenced or altered by the drumming block immediately before it (see Fig. 1).

For each frequency of interest (1.5 Hz, 4 Hz, and 9.5 Hz) and each drumming condition (1.5-bps, 4-bps, and 9.5-bps), we calculated the percent change in rhythmicity during the drumming compared to the baseline using the following formula:

$$\:Coupling\:Score\:\left(\%\right)=\:\frac{{LAVI}_{Drumming}-\:{LAVI}_{S1}}{{LAVI}_{S1}}*100$$

Here, $\:{LAVI}_{Drumming}\:$represents the rhythmicity value during the drumming section, and ​$\:{LAVI}_{S1}$ is the rhythmicity value during the baseline (S1). A higher Coupling Score reflects greater rhythmic alignment between the EEG activity and the external drumming stimulus, indicating stronger neural tracking.

Coupling scores greater than 3 standard deviations from the mean were considered outliers and were removed from analysis. Two observations were removed from any analysis related to the coupling scores (1.5 bps, z = 3.2, z = 3.15).

It should be noted that there was a significant difference between the distribution of coupling scores at 1.5 Hz (mean = 33.3, Sd = 28.5), 4 Hz (mean = 11.4, Sd = 16.91), and 9.5 Hz (mean = − 6.7, Sd = 13.09). These are expected when comparing activity across different bands, and might reflect inherent differences between these bands (see discussion).

## Results

To elucidate the neural mechanisms underlying drumming-induced altered states of consciousness (ASC), we measured subjective altered experience (AE) using a validated questionnaire, assessed subjective time perception, and recorded EEG during three distinct blocks of drumming. Each drumming block employed a different rhythm, corresponding to a unique neural oscillatory band (delta (1.5-bps), theta (4-bps) and alpha (9.5-bps)). Our hypothesis was that higher neural rhythmic tracking, specifically at theta (4-bps drumming) would correspond to a stronger ASC experience. Before delving into the EEG and the relationship between neural tracking and subjective experience, we first examined whether there was a general effect of the drumming rhythms on the subjective reports, and whether it was specific to 4-bps drumming.

We began by assessing the difference in AE, as measured by the questionnaire (see "Methods"), across drumming conditions. We conducted a repeated-measures ANOVA with the drumming rhythm as a within-subject factor (levels, 1.5, 4, and 9.5 bps). We found a significant main effect of drumming rhythm on AE (F(2,76) = 3.1, *p* = 0.05). Since our hypothesis predicted the 4-bps drumming to induce the strongest AE, we performed post-hoc paired t-tests, comparing the AE scores during the 4-bps drumming to the other rhythms. Contrary to expectations, AE scores were not significantly higher during 4-bps drumming compared to 1.5-bps (t(38) = 0.42, *p* = 0.67) or 9.5-bps (t(38) = − 1.79, *p* = 0.081). However, a significant difference emerged between the 9.5- and 1.5-bps conditions, with higher AE scores reported during 9.5-bps drumming (t(38) = 2.43, *p* = 0.02). These findings suggest that, based purely on self-reported subjective experience, 9.5-bps drumming induced stronger altered experience than 1.5-bps drumming, while the theta-range (4-bps) rhythm did not produce significantly different AE scores relative to the other conditions.

To examine whether drumming affects time estimation, we used a similar repeated-measures ANOVA, treating subjective time estimation as the dependent variable and drumming rhythm as the within-subject factor. Contrary to our hypothesis, no significant effect of rhythm on time estimation was found (F < 1). This suggests that, on average, no specific drumming rhythm consistently influenced participants’ perception of elapsed time.

Lastly, we examined the relationship between the two subjective measures, time estimations and AE scores. To account for between-subject variability in this within-subject design, each participant’s AE scores and time estimates were normalized by their individual mean. Consistent with our prior assumption we found a significant correlation between the two measures (rho = − 0.18, *p* = 0.04). We report here (and in subsequent analyses involving these measures) spearman correlation due to the ordinal nature of AE and the non-linear, and scalar property of time estimation^42,43^.

Together, these results indicate that, contrary to our hypothesis, 4-bps drumming did not lead to increased AE scores, or prolonged time estimations compared to the other drumming conditions. Nevertheless, it remains possible that individual differences in neural responses to the drumming could account for the lack of a group-level effect. Next, we examined the EEG and its relationship to the subjective experience.

### Validating neural tracking in the EEG data

Before testing our main hypothesis, whether stronger neural rhythmic tracking leads to stronger ASC, we turned to establish a reliable neural tracking of the drumming rhythms. To assess EEG rhythmicity, we quantified rhythmicity using a phase-base method (LAVI, see "Methods") across frequencies (Fig. 3a). To initially assess rhythmic tracking, we used paired t-tests to examine the differences in the LAVI scores at each drumming frequency during the drumming compared to baseline (S1). We used FDR p-value correction for multiple comparisons to control for false discovery rates. The LAVI values were significantly higher during drumming compared to baseline at 1.5 Hz (t(38) = 8.03, p(FDR) = 3e-9, Cohen’s d = 1.87, 95% CI [1.10, 2.64]) and 4 Hz (t = 4.01, p(FDR) = 3e-4, Cohen’s d = 0.93, 95% CI [0.38, 1.49]). At 9.5 Hz, the LAVI scores were significantly lower during drumming compared to baseline S1 (t(36) = − 4.28, p(FDR) = 1e-4, Cohen’s d = − 0.34, 95% CI [-0.5, − 0.18]). We then computed our main dependent variable - a coupling score by comparing rhythmicity during drumming to the preceding silent baseline (S1). To test for rhythm-specific tracking, we conducted one-tailed paired t-tests comparing coupling scores at each rhythm’s corresponding frequency (e.g., 1.5 Hz brain rhythm during 1.5 bps drumming) against the same brain frequency during the other drumming conditions (see Fig. 3b.). We used an additional FDR p-value correction for multiple comparisons as previously. As expected, coupling scores at 1.5 Hz were significantly higher during the congruent 1.5-bps drumming than during 4-bps (t(38) = 5.56, p_(FDR)_ = 3e-6) and 9.5-bps drumming (t(38) = 6.59, p_(FDR)_ = 3e-7). Similarly, at 4 Hz, coupling scores were significantly higher during 4-bps drumming than during 1.5-bps drumming (t(38) = 2.33, p_(FDR)_ = 0.018) and 9.5-bps drumming (t(38) = 2.73, *p* = 0.009). At 9.5 Hz, however, no significant difference in coupling was found during 9.5 and 1.5-bps drumming (p_(FDR)_ = 0.5). Similarly, no significant difference was found in this frequency between 9.5 and 4-bps (t(36) = 1.6, p_(FDR)_ = 0.07). These findings demonstrate robust neural tracking during the drumming, suggesting rhythmic tracking, in the 1.5 and 4 bps drumming conditions, but not in the 9.5-bps condition (see discussion).

### Neural tracking predicts subjective altered experience but not time distortion

Having established neural rhythmic tracking for 1.5-bps and 4-bps drumming, we next tested our primary hypothesis: that rhythmic tracking to the 4-bps drumming rhythm, corresponding to theta, would be associated with heightened altered experience (AE) reports and altered time perception.

**Fig. 4:**
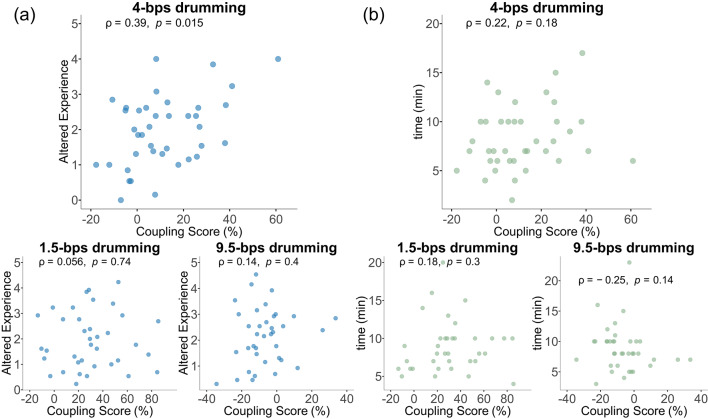
Associating neural rhythmic tracking to altered experience and time estimation. **a** Spearman correlation between coupling scores during each of the drumming conditions and the respective altered experience scores (AE) scores. A rhythm-specific effect was found at theta rhythm (4-bps). **b** Spearman correlation between coupling scores during each of the drumming conditions and the respective time estimations of the participants. No significant relationship was found between time estimations and coupling.

We began by examining the effect of neural tracking (i.e., coupling scores) on AE. We used a Spearman correlation between the coupling scores at 4 Hz and the AE scores during 4-bps drumming (Fig. 4a). A significant positive correlation was found (rho = 0.39, *p* = 0.01). This indicates that greater neural tracking at theta was associated with stronger reports of altered experience, supporting our hypothesis. To examine the specificity of this effect, we repeated the analysis for the other drumming conditions. No significant relationships were found for coupling at 1.5 Hz and the AE scores during 1.5-bps drumming (rho = 0.056, *p* = 0.74). Similarly, no significant relationship was found for the 9.5-bps drumming (rho = 0.14, *p* = 0.4), emphasizing the unique association of neural tracking and AE at theta supporting our hypothesis.

We then examined whether a similar relationship exists between the time estimations and coupling scores (Fig. 4b). Here, we examined the correlation between 4 Hz coupling scores time estimation of the 4-bps drumming condition. Contrary to our predictions, this relationship was not significant (rho = 0.22, *p* = 0.18). Likewise, no significant correlations were observed in the 1.5-bps (rho = 0.18, *p* = 0.3) or 9.5-bps (rho = − 0.25, *p* = 0.14) conditions. Overall, neural tracking did not predict time perception of drumming duration at any rhythm tested.

Finally, we examined whether coupling at 4 Hz predicted participants’ average AE scores across other drumming conditions. We found a significant correlation (rho = 0.32, *p* = 0.04), suggesting that individuals with stronger theta-band tracking during 4-bps drumming also tended to report higher AE overall, regardless of.

the drumming condition. This relationship with the mean AE score was limited to the 4 Hz coupling. No significant associations were observed for the neural brain coupling to the 1.5-bps (i.e., 1.5 Hz, rho = − 0.01, *p* = 0.95) or 9.5-bps (i.e., 9.5 Hz, rho = − 0.01, *p* = 0.92) drumming. When recalculating the average AE based on reports following the 1.5 and 9.5-bps drumming only (excluding 4-bps) the correlation between the mean AE in these conditions and the coupling at theta was still trending (rho = 0.29 *p* = 0.08). This suggests that theta brain coupling to 4-bps drumming might be informative in understanding susceptibility to ASC. Future studies, targeting this effect, would be required to further validate this possibility. Theta coupling scores did not predict participants’ average time estimations in any drumming condition (1.5 bps: rho = 0.18, *p* = 0.29; 4 bps: rho = 0.14, *p* = 0.39; 9.5 bps: rho = − 0.06, *p* = 0.72).

In summary, our findings indicate that while on a group level, 4-bps drumming rhythm did not elicit heightened AE or subjective time distortion, AE was modulated by differences in neural tracking at the theta frequency. Specifically, stronger theta-band tracking during 4-bps drumming predicted higher AE. Additionally, theta-band neural tracking during 4-bps drumming was also positively correlated with participants’ average AE scores across all drumming conditions, indicating that individuals with stronger theta entrainment at 4 Hz tend to experience higher AE in general. These findings support the notion of a rhythm-specific neural mechanism at theta underlying altered states of consciousness.

## Discussion

In this study, we investigated whether neural rhythmic tracking, specifically at theta (4 Hz), could evoke subjective experiences of altered states of consciousness (ASC). Although self-reported ASC measures alone did not significantly differ between the 4-bps condition and the other rhythms (1.5 and 9.5-bps), integrating these reports with EEG data revealed a nuanced pattern: the variability in participants’ subjective experience following 4-bps drumming was mediated by the degree of neural tracking to this rhythm. Specifically, while robust rhythmic coupling was observed at both 1.5 and 4-bps, only the theta-band coupling at 4 Hz was significantly associated with increased AE scores. This rhythm-specific association supports the notion that theta activity plays a unique role in the facilitation of altered states. The absence of a similar association at 1.5 Hz, despite strong neural coupling, emphasizes the functional specificity of the theta range in modulating subjective experience. These findings align with our hypothesis, and could explain the cross-cultural usage of these rhythms in trance-related ceremonies. By using novel and rigorous measurements of EEG rhythmicity, and integrating individual differences in the EEG response to the drumming, our findings settle previous contradicting findings on the effect of theta related drumming on ASC.

Another intriguing rhythm-specific result was that 4-Hz coupling not only predicted AE within the 4-bps condition but also correlated with participants’ average AE scores across all drumming conditions. Individuals with stronger neural tracking at 4 Hz appeared more prone to altered states regardless of the rhythm, pointing to a possible biomarker of altered state susceptibility. In contrast, neither 1.5–9.5 Hz coupling predicted AE across conditions, further reinforcing the specificity of theta-band tracking in accounting for subjective alterations. This suggests that theta neural tracking may reflect a broader trait-like susceptibility to ASC, regardless of the specific external rhythmic environment. The possibility that 4-Hz coupling could serve as a biomarker for ASC susceptibility is particularly compelling. Developing a biologically grounded, objective index for altered state proneness could significantly advance both experimental paradigms and therapeutic applications. Similar findings were found in hypnosis, a closely related ASC to trance, where endogenous frontal theta was higher in highly susceptible individuals^44,45^. Nevertheless, as far as we know, this study is the first to demonstrate a connection between externally induced theta and ASC susceptibility. Future studies should investigate the potential of theta tracking in discerning ASC susceptibility, by examining the stability of 4-Hz coupling across contexts and its relation to individual differences in ASC responsiveness.

This work focuses on the brains’ response to the drumming stimuli. We referred to this as neural tracking and refrained from directly inferring whether the drumming at each frequency entrained an internal oscillator or instead elicited a rhythm through successive evoked responses. Regardless of the neurophysiological mechanism resulting in this measure, which remains an open question, the magnitude of the measured neural tracking at 4 Hz related to the degree of altered states reported by the participants. Future studies can attempt discerning whether true entrainment of internal oscillators accounts for the link between neural tracking and altered state or whether it is, in fact, the evoked rhythmic responses.

Theta oscillations have been related to a range of cognitive and neurophysiological networks and processes that potentially overlap with the phenomenology of ASC, offering several plausible mechanistic explanations for this link. First, structurally, theta activity is strongly associated with areas in the prefrontal cortex (e.g., medial PFC (mPFC) and dorsolateral PFC (dlPFC)) and hippocampal-prefrontal networks, which are associated with ASC phenomenology. These include self-referential processing^46,47^, memory integration^48^, spatial working memory^49^, and executive control functions^50^. Theta rhythms in these prefrontal networks may serve as an important mechanism for information integration^51^. The positive anterior cluster in the theta coupling-scores we describe is consistent with the idea that endogenous theta phase-coupling in these areas is modulated - either facilitated, overridden, or disrupted - by external rhythmic stimuli. Previous work support this idea by showing that modulating theta in the PFC exogenously via transcranial alternating current stimulation (tACS) can indeed alter cognitive performance in different task domains, such as memory performance, motor imagery (i.e., mental simulation of a motor act) and visual recognition and encoding^52–54^. Furthermore, abnormalities in theta coupling in these networks have been associated with various psychopathological disorders such as schizophrenia^55–57^, complex PTSD^58^, and major depression^55^. These conditions share several phenomenological features with ASC, such as dissociation disorders, hallucinations, loss of bodily and executive control and distorted time perception^59^. This further supports the idea that similar neural structures and networks are engaged both in psychopathological conditions and in ASC - with the key distinction that, during ceremonial or ritual contexts, these neural dynamics are intentionally and safely invoked by the practitioner. This parallel opens avenues for investigating the therapeutic potential of rhythmically induced ASC. To the best of our knowledge, there was only one functional magnetic resonance imaging (fMRI) study during rhythmically induced trance states, which could help support these structural claims^24^. While this study did not show increased activity in the hippocampus or mPFC during trance compared to resting state, it did show enhanced activity in the dlPFC. Further neuroimaging and electrophysiological studies are required to clarify the functional neuroanatomy of ASC and its relation to theta activity.

Another possible explanation of the association between ASC and theta is the role of theta oscillations in facilitating long-range communication between distant brain regions, promoting synchronization and integration across networks^60,61^. In this sense, increased theta power during ASCs may reflect a shift in top-down hierarchical control and a reconfiguration of information integration, aligning with models like the “entropic brain hypothesis"^62^. This model proposes that ASCs are marked by increased entropy, meaning more unstructured brain functions, leading to a decrease in the established modular segregation and a disruption of the normal hierarchical integration characteristic of everyday consciousness. It could then be possible that strong theta tracking facilitates these states of endogenous synchronization, contributing to the cognitive and experiential qualities of ASC. Taken together, these insights suggest that theta oscillations may constitute a central neural mechanism in the emergence of ASC. Nevertheless, the precise way in which endogenous and exogenously-driven theta may interact in relation to ASC remains an open and important question for future research.

In addition to the explicit self-report questionnaire, we also explored a putative connection between ASC and an estimation of elapsed time. We report a correlation between time estimations and AE scores. Although time distortions are subjectively included in self-report measures of AE, our findings lay the foundations of future work investigating whether temporal decisions might interestingly complement traditional subjective measures of ASC. If a consistent relationship between subjective time and altered states can be demonstrated in future work, time estimation tasks could provide a simple and implicit tool for assessing ASC. Identifying and validating implicit and behavior-based measures, such as time estimation tasks (or other perceptual and cognitive distortions associated with ASC) would greatly enrich the methodological toolkit of ASC research, which heavily relies on subjective reports. Nevertheless, unlike AE, time estimation was not modulated by the level of neural tracking at any rhythm. Further research is needed to determine the exact methodology and reliability of time-based tasks for capturing altered states, and its’ possible relationship to neural tracking.

The current study has a few notable limitations we discuss next. Although the choice of drumming frequencies is well motivated by the literature, we were unable to adequately estimate neural tracking to 9.5 bps drumming. The lack of clear characterization of neural tracking in this rhythm precluded our ability to examine the association between neural tracking at this frequency and subjective experience. Previous work has shown that that alpha is relatively stable and resistant to external modulation, making them difficult to externally modulate^63^. Thus, it is possible that internally generated alpha rhythms, which predominate the electrophysiological response in these frequencies, functionally disrupted our ability to measure neuronal tracking for 9.5 bps drumming. This account is of particular relevance seeing that participants were instructed to close their eyes whilst listening to the drumming - a condition well known to further promote internally generated alpha band oscillations. At an experimental level, it is worth considering running future studies with participants instructed to keep their eyes open. Interestingly, tailoring the external rhythmic stimulation to each participant’s unique alpha frequency has been shown to enhance the modulation of intrinsic alpha frequencies^64,65^. Future work might consider accounting for individual spectral profiles. It is possible that tailoring the drumming rhythms to individuals’ delta, theta and alpha bands could both promote the neural tracking of said rhythmic activity and lead to enhancing more functionally relevant brain processes - leading to greater modulation of neural tracking and subjective experience. Altogether, these insights highlight a promising direction: the development of individualized rhythmic stimulation protocols aligned with endogenous spectral dynamics.

Lastly, the present study recruited participants inexperienced in ASC. Although we anticipated considerable variability in susceptibility to ASC, we could only assess this variability post-hoc. A valuable extension of this work would involve recruiting experienced practitioners and a matched control group. Since some of our inexperienced participants didn’t have any significant subjective experience shifts in either of the drumming conditions due to lack of susceptibility or engagement, it could be possible that an experienced group would show different, or perhaps stronger effects. More broadly, progress in understanding the neural mechanisms of altered states of consciousness depends on developing simple, reliable tools for assessing individual susceptibility - tools that are currently lacking. For example, personality measures show no clear relationship to hypnotic responsiveness^66^, and psychedelic research similarly finds considerable uncertainty in how personal traits predict responses^67^. The absence of such measures for drumming induced trance underscores the practical need for a biomarker of susceptibility. Future studies should test whether neural tracking of theta provides such a biomarker, as suggested by our results, potentially allowing susceptibility to be assessed prior to experimentation and enable more targeted recruitment.

In conclusion, this study sheds light into the neurophysiological mechanisms underlying rhythm-induced ASC. By combining EEG-based measures of rhythmic coupling with subjective reports, we demonstrated a theta rhythm-specific relationship between neural dynamics and altered experience and identified theta coupling as a potential biomarker for ASC susceptibility. This association between 4 Hz coupling and altered experience, both within and across conditions, points to theta tracking as a promising candidate for future ASC research.

Thus, our findings form a foundation for future studies to refine the use of rhythmic stimulation as a tool for investigating ASC and to broaden the methodological approaches available for capturing these complex experiences. This research contributes to the growing effort to relate brain dynamics with subjective phenomena, an essential step toward a more integrative and empirically grounded science of consciousness. Through this interdisciplinary bridge between a prevalent cultural practice and cognitive neuroscience, our work suggests that ritual drumming is not merely symbolic, but may be rooted in real, measurable neural mechanisms underlying ASC and perhaps consciousness as a whole.

## Supplementary Information

Below is the link to the electronic supplementary material.

Supplementary Material 1

## Data Availability

The data supporting the findings of this study are available from the corresponding author upon reasonable request. Requests can be directed either to Yoel Gordon at yoel.gordon@mail.huji.ac.il, or to Prof. Ayelet Landau at ayelet.landau@gmail.com.
