# Supplementary material for: Neural tracking at theta predicts drumming-induced altered states of consciousness
Source: Sci Rep. 2026 Mar 26;16:10204. doi: 10.1038/s41598-026-37700-x (PMC13022407; doi:10.1038/s41598-026-37700-x)
Supplement: Supplementary file 1 — Supplementary Material 1 [file 41598_2026_37700_MOESM1_ESM.pdf]

Supplementary

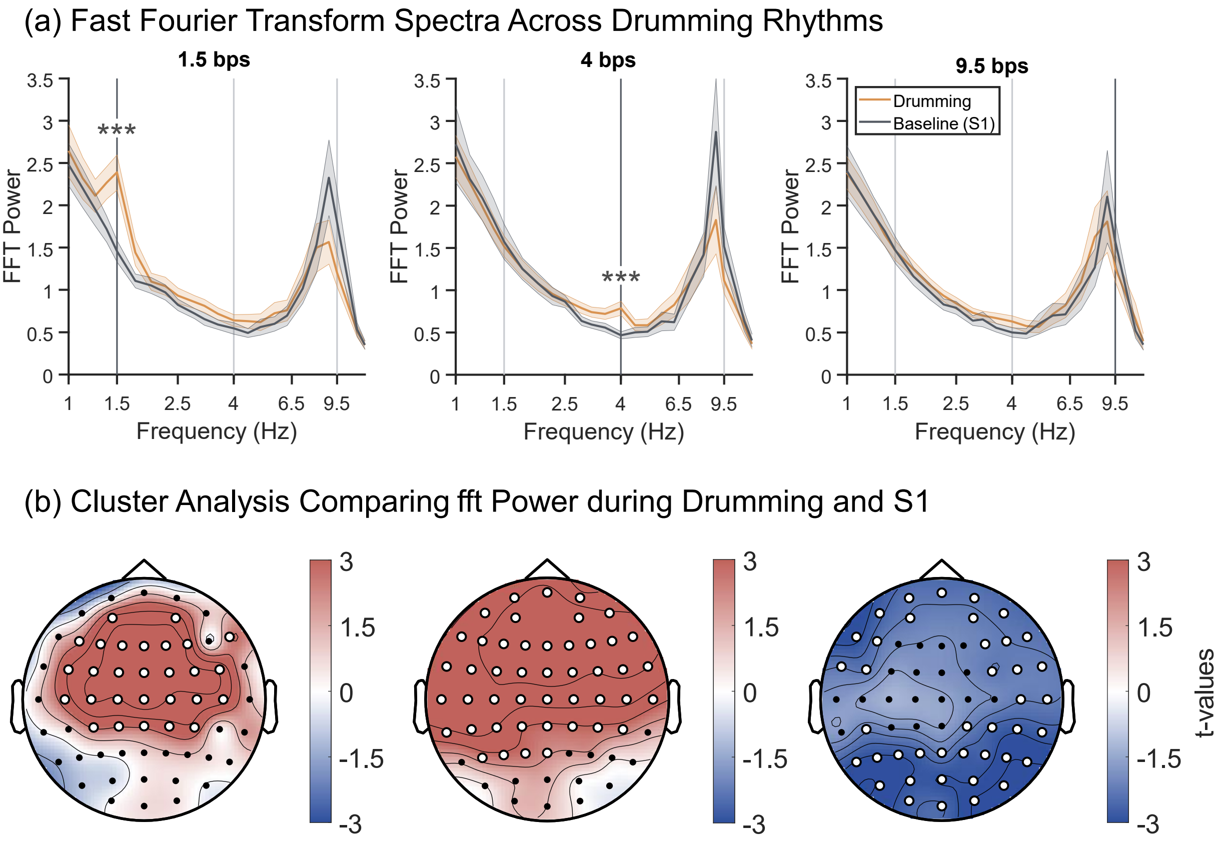

Supplementary figure 1: Power Spectra During Drumming and Baseline (S1) in the Three Drumming Rhythms.

(a) Power assessed using a standard Fast Fourier Transform (FFT). To complement our novel phase-based metric, we provide here a widely-used FFT analysis. We computed the FFT (using ft_frequencyanalysis) on Hann windowed across a logarithmically spaced frequencies of interest. We used paired t-tests to compare the power during drumming and S1 in each frequency. Similar to the main rhythmicity spectra displayed in figure 3, drumming at 1.5 and 4 Hz resulted in a significant peak at the corresponding neural frequencies.

(b) Topographies of the cluster analysis comparing power during drumming and S1 (baseline). These were generated using a Monte Carlo cluster analysis with 1000 repetitions. Topographies plot averaged power within a ±0.2 Hz window across all participants (1.3-1.7 Hz, 3.8-4.2 Hz, and 9.3-9.7 Hz respectively). Note the scale differences between conditions in the topographies.

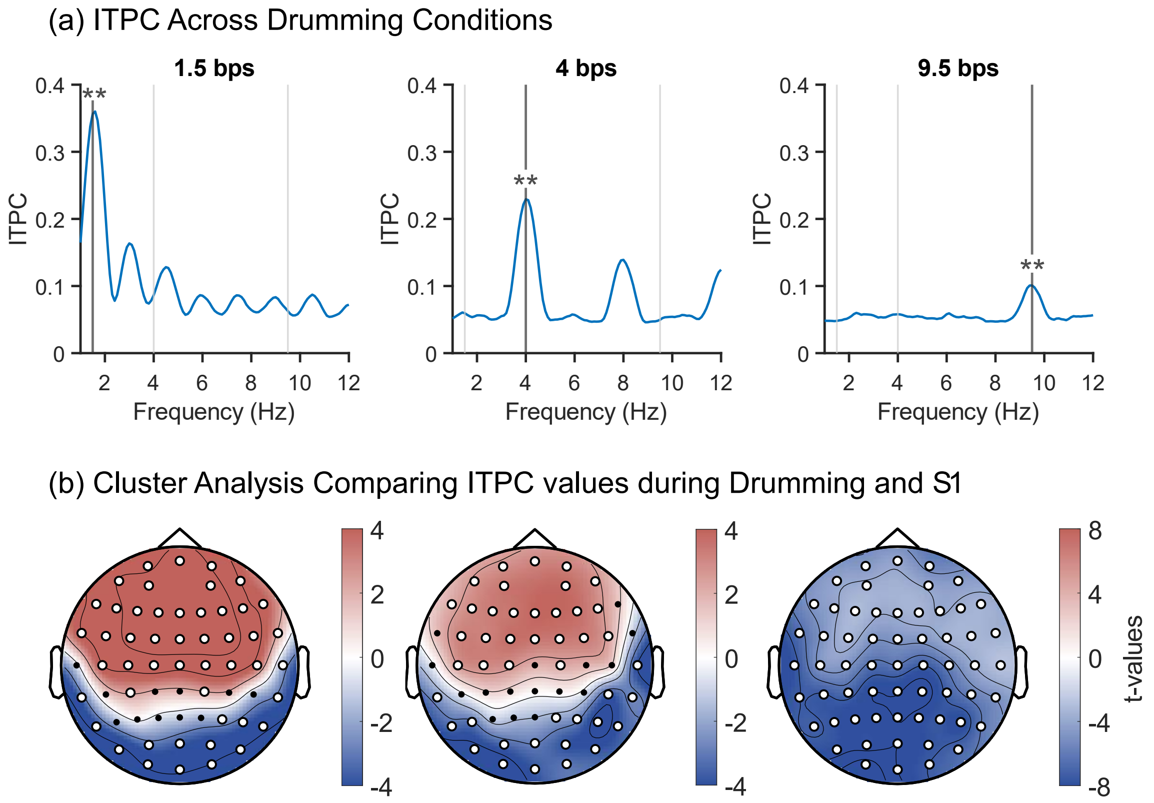

Supplementary figure 2: Inter-Trial Phase Coherence (ITPC) Across the Different Drumming Conditions.

(a) Inter Trial Phase Coherence spectrum. We segmented our data into 2-second trials on non-overlapping trials centered on auditory drum beats. We then computed ITPC for each drumming condition separately. We conducted permutation test with a similar amount of randomized 2 second windows (i.e., not beat-locked) for each participant and calculated the mean ITPC across-participants. We repeated this process 500 times for each frequency. Our results reveal significant phase coherence at frequency corresponding to each external drumming rhythm.

(b) Topographies of the cluster analysis comparing ITPC during drumming and S1 (baseline). These were generated using a Monte Carlo cluster analysis with 1000 repetitions. Topographies plot averaged ITPC values within a ±0.2 Hz window across all participants (1.3-1.7 Hz, 3.8-4.2 Hz, and 9.3-9.7 Hz respectively). Note the scale differences between conditions in the topographies.
